# The peri-electrode space is a significant element of the electrode–brain interface in deep brain stimulation: A computational study

**DOI:** 10.1016/j.brainresbull.2007.07.007

**Published:** 2007-10-19

**Authors:** Nada Yousif, Richard Bayford, Peter G. Bain, Xuguang Liu

**Affiliations:** a Department of Clinical Neuroscience, Division of Neuroscience and Mental Health, Faculty of Medicine, Imperial College London, UK; b The Bio-Modelling/Bio-Informatics Group, Department of Biomedical Science, Institute of Social and Health Research, Middlesex University, London, UK; c The Movement Disorders and Neurostimulation Unit, Department of Neuroscience, Charing Cross Hospital, London, UK

**Keywords:** Deep brain stimulation, Electrode–brain interface, Structural model, Finite element method

## Abstract

Deep brain stimulation (DBS) is an increasingly used clinical treatment for various neurological disorders, particularly movement disorders such as Parkinson's disease. However, the mechanism by which these high frequency electrical pulses act on neuronal activity is unclear. Once the stimulating electrode is placed *in situ*, an electrode–brain interface (EBI) is created. To compensate for the lack of studies on the effects of this generic depth EBI on therapeutic DBS, we constructed a three-dimensional computational model of the EBI using the finite element method, in which the structural details and biophysical properties of the EBI are preserved. Our investigations focus on the peri-electrode space as a significant element of the EBI, and its physiological and pathological modulation, in particular by brain pulsation and giant cell formation. We also consider the difference between the current fields induced by different configurations of the quadripolar electrode contacts. These results quantitatively demonstrated that the peri-electrode space is a significant element of the EBI and its biophysical properties are modulated by brain pulsation and giant cell formation, as well as by the choice of electrode contact configuration. This study leads to a fuller understanding of the EBI and its effects on the crossing electric currents, and will ultimately lead to optimisation of the therapeutic effects of DBS.

## 1 Introduction

Deep brain stimulation (DBS) is an increasingly used therapy for the treatment of a number of neurological disorders [8,15,17,25], which involves the uni-lateral or bi-lateral implantation of multi-contact electrodes into condition-specific targets of the brain. Once the electrode is implanted in a deep brain region, an electrode–brain interface (EBI) is formed, which the stimulating current must cross in order to reach the neural target. Therefore, the biophysical properties of this interface will affect the therapeutic outcome of stimulation. However, there is presently no method of investigating the depth EBI as it is impossible to directly measure or visualise the spread of current in the stimulated region in the human brain.

The purpose of this research is to compensate for the lack of such quantitative investigations, by developing a biologically based three-dimensional structural model of the EBI based on the finite element method (FEM [28]). This model consists of three main structural components: (i) the implanted electrode; (ii) the surrounding brain tissue; (iii) a layer of peri-electrode space surrounding the electrode, which is filled with extracellular fluid at the acute stage during and immediately after implantation. A similar computational approach has been used in previous studies, however in these studies the peri-electrode space was not specifically defined [12,13,18].

Simulations of the structural EBI model are based on our previous *in vitro* morphological and *in vivo* physiological studies, in which we have demonstrated that the biophysical properties of the peri-electrode space evolve over time. It was observed that giant cell type reactions occur at the surface of implanted DBS electrodes [19], and were present irrespective of the duration of implantation, and as early as 2 weeks post-implantation, as seen in other studies [2]. Furthermore, by recording through the implanted electrode *in situ*, we observed a low-frequency electrode potential across four different brain regions, and four neurological conditions [27]. This LFP component was modulated in frequency and amplitude during mild exercise, and this modulation was correlated to that of the simultaneously recorded blood pressure signal. These results suggest that the electrode potential is influenced by brain pulsation, which disturbs the electric charge on the electrode surface, and induces a measurable a potential change [27].

In the current study we present a three-dimensional model of the EBI containing the peri-electrode space and simulate our previous studies [19,27]. This model is based on the generic features of the peri-electrode space in the depth EBI, which are independent of the structure of the surrounding brain tissue specific to the electrode location, the neurological disorder treated by stimulation, and of the instrumentation used. We used this model to focus on the specific influence of the peri-electrode space on the electric field induced by DBS. In particular, the effects of: (i) the physiological modulation of the EBI due to brain pulsation; (ii) the pathological formation of giant cells; (iii) different contact configurations of the quadripolar electrode, are quantified to demonstrate that the inclusion of the peri-electrode space in the EBI model is critical, in particular when considering the distribution of the stimulation-induced electric field during acute versus chronic stages of DBS.

## 2 Materials and methods

### 2.1 The electrode–brain interface and its structural FEM mode

**Fig. 1:**
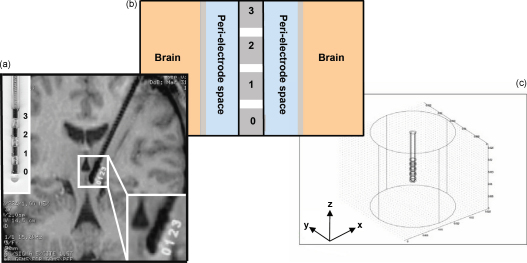
Structural definition of the electrode–brain interface (EBI): (a) an MRI of the implanted quadripolar DBS electrode *in situ* with enlargements of the electrode (left upper corner) and the electrode–brain interface (right lower corner), (b) schematic representation of the EBI consisting of the implanted electrode with an array of four contacts (numbered 0–3), the surrounding neural tissue, and the “peri-electrode space” in between, and (c) the structure of the three-dimensional FEM model, as created in COMSOL multiphysics. This structural model takes into account the detailed geometry of the electrode (Medtronic model 3389), a peri-electrode space of 0.25 mm in thickness, and a cylinder of neural tissue of 10 mm in radius.

We defined the EBI based on the generic structural composition of the interface, as seen in histology images of the region around implanted depth electrodes [19,21], and in a post-mortem examination of the stimulation site in a DBS patient case study [3] (Fig. 1a). Hence, the EBI consists of: (i) the implanted DBS electrode(s) based on the manufacturer's description of the quadripolar electrode (model 3389, Medtronic, MN) (Fig. 1b); (ii) the surrounding brain tissue; (iii) a layer of peri-electrode space surrounding the implanted electrode, which is filled with extracellular fluid in the acute stage, and reactive cells in the chronic stage. The modelling package COMSOL Multiphysics 3.3 [7] was used to create a three-dimensional FEM model of the interface (Fig. 1c). The finite element method allows us to model a three-dimensional structure by discretising it into smaller volumes, in order to estimate the solution to a partial differential equation, which describes the electric field distribution over a bounded volume [28]. The volume was meshed into 119,512 tetrahedral elements using a Delaunay meshing algorithm [7] within COMSOL, and further increasing the number of elements did not improve the accuracy of the solution. The peri-electrode space was arbitrarily defined as 0.25 mm thick, in order to be wide enough to allow the FEM mesh in this layer to contain up to four elements and ensure the accuracy of the solution, while remaining small compared to the dimensions of the electrode and surrounding tissue. Halving or doubling this thickness creates an average of <2% difference in the percentage changes between conditions.

The potential distribution induced by stimulation was calculated by solving the Poisson equation, and assuming that there are no internal current sources within the bounded region:

∇⋅σ∇V=0

where *V* is the potential (measured in V), and *σ* is the conductivity (measured in S/m). The mean conductivity values of the brain tissue were defined based on previous biological studies. As we focus on the peri-electrode space, we did not account for detailed anatomy of the brain, and hence the surrounding brain tissue was modelled as a homogenous cylinder of grey matter, with conductivity as *σ*_tissue_ = 0.2 S/m [11]. To simulate the EBI at the acute stage, during which the peri-electrode space is filled by extracellular fluid (ECF), we used the value of *σ*_ECF_ = 1.7 S/m [23] for this layer. These values [10] are robust, as doubling or halving conductivity values only creates an average difference of <2% between percentage changes across conditions.

In order to simulate the injected current, the active contacts were set to the desired stimulating potential in volts, and the outer boundary of the cylinder of surrounding tissue (which is 10 mm in distance from the surface of the electrode) was constrained to 0 V via Dirichlet boundary conditions. For monopolar stimulation this boundary condition represents the stimulator case being grounded to zero volts, as in clinical practice. For bipolar stimulation, electrode contacts are activated with opposite polarity. The choice of Dirichlet boundary conditions at the outer boundary did not affect the field shape in the bipolar case, and affected the absolute value of the potential only by ∼1%. The non-active contacts and insulating parts of the implanted electrode were bound using Neumann conditions, which constrain the derivative of the electric potential through these boundaries to be zero.

### 2.2 Physiological modulation of the EBI

We examined the modulation of the induced potential distribution by brain pulsation, as shown by our previous physiological study [27]. We assumed that as blood flows into the brain in the acute stage post-implantation, two effects occur on the biophysical properties of the EBI:

1. Change in the conductivity of the surrounding tissue, as blood has higher conductivity than brain tissue.
2. As blood perfuses into the brain, the intra-cranial pressure increases, as does the pressure on the electrode surface. Furthermore, during electrode implantation an uneven distribution of pressure may occur as a result of craniotomy. In this case, the ECF in the peri-electrode space may flow to a region of lower pressure along the electrode trajectory towards the burr hole, and therefore the thickness of the peri-electrode space may change. Changes in both pressure on the electrode surface and thickness of the peri-electrode space will alter the biophysical properties of the electrode–brain interface.

We take a combination of these two factors into account, and quantified these changes as follows:

1. The intra-cranial volume and pressure changes were calculated based on the calculations of Alperin et al. [1], using magnetic resonance imaging (MRI) measurements of cerebrospinal fluid (CSF) and blood flow. In their study, the volume change was calculated from the net transcranial CSF and blood volumetric flow rates, which gave a change of between 0.3 and 1.3 ml in total brain volume per cardiac cycle in healthy volunteers. In the present study, to simulate the effect that brain pulsation may have on the electrode–brain interface, we used the value of 1.3 ml or 0.09% (1.3 ml/1400 ml) to represent the total volume change for a closed cranium, and to simulate the maximally possible effect of brain pulsation on the EBI in an opened cranium during electrode implantation.
2. The brain consists of 16% extracellular fluid (ECF). The rest is grey and white matter in a 2:3 ratio, such that grey matter constitutes 33.6% and white matter constitutes 50.4%. Impedance of grey matter plus 16% ECF: (0.16 × 60) + (0.84 × 350) = 303.6. Plus the perfused in volume of blood: (0.0009 × 150) + (0.16 × 60) + (0.84 × 350) = 303.735. This is a 0.04% change in the impedance, and conductivity.
3. This value yields a change in conductivity from 0.2000 S/m to a maximum of 0.2001 S/m.
4. Finally, when blood perfuses into the brain, the 0.09%, increase in brain volume by may induce an equivalent volume change of the peri-electrode space, which would result in a 0.12% decrease in the thickness from 0.250 to 0.247 mm.

### 2.3 Giant cell reactions

Accounting for the pathological growth of giant cells at the electrode surface [19], it was assumed that the conductivity of this giant cell layer in chronic stages will be much lower than that of ECF and of grey matter and therefore we set the conductivity of the peri-electrode space to 0.125 S/m, which is equivalent to the reported values for white matter. This is comparable with values used for the encapsulation layer in previous modelling studies [18].

### 2.4 Electrode contact configuration

The stimulation settings commonly used in clinical practice are monopolar and bipolar configurations, and we simulated the electric fields induced by these settings, while keeping the overall potential difference applied in all simulations identical. We examined what effect (i) the inclusion of the peri-electrode space and (ii) the changes within this space, have on the fields induced by monopolar and bipolar settings.

### 2.5 Quantification

The simulation results are presented as the potential distribution (*V*) as obtained directly from Poisson's equation, or as the electric field (*E*) distribution, where *E* is calculated by the following equation:

E=−∇V

The electric field *E* is a measure of the how the electric potential changes over space, and is proportional to the current density. Note that *E* is a three-dimensional vector and was plotted using its ‘norm’, which is calculated, using the standard definition for the norm of a vector:

$$E_{\text{norm}}=\sqrt{\left(E_{X}^{2}+E_{Y}^{2}+E_{Z}^{2}\right)}$$

In order to quantify the difference between contact settings, the percentage difference between average potential values was quantified using the following equation:

$$\text{percentage}\;\text{change}=\left(\frac{\text{value}\;2-\text{value}\;1}{\text{value}\;1}\right)\times 100\%$$

The electric field was sampled at 120 intervals in a radius around contact 0, and the field was averaged within each 30° phase to give 12 measurements (360°) of average field strength. We calculated the percentage change of field strength between conditions and present these results with ±1 standard deviation. We used a two-way analysis of variance (ANOVA) to quantify the difference in percentage changes between content of the peri-electrode space (homogenous tissue, ECF, or giant cells), and the electrode configuration (monopolar or bipolar).

## 3 Results

### 3.1 The EBI with and without the peri-electrode space

**Fig. 2:**
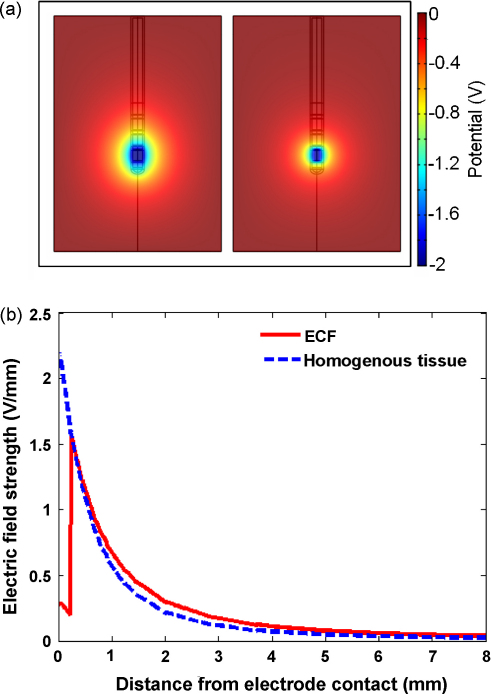
This figure shows the difference in the (a) potential distribution when the peri-electrode space surrounding the implanted electrode is filled with fluid (left) or forms part of the homogenous surrounding tissue (right). (b) The cross-section through the electric field distribution in the ECF (solid line) and homogenous tissue (dashed line) cases. These cross-sections are measured radially outwards from the surface of contact zero. The figures show that the induced potential distribution spreads further into the tissue when there is a ECF-filled peri-electrode space included in the model.

Our current model differs from previous theoretical approaches by including and focusing on the peri-electrode space, in order to examine what effect the inclusion of the peri-electrode space has on the potential distribution induced by DBS. Compared with an EBI without a peri-electrode space, i.e., the electrode is surrounded by homogenous brain tissue (right half, Fig. 2a), the ECF layer allows the potential to spread further over distance (left half, Fig. 2a). This is intuitive, as the conductivity of extra-cellular fluid is higher than that of grey matter, and therefore the ECF provides a path of least resistance through which current flows. The electric field strength attains a 40% decrease at the fluid-tissue interface when the ECF layer is present, compared with the equivalent measurement when the ECF layer is discarded. This can be further demonstrated by the cross-section curves in Fig. 2b. In the ECF case (solid line) this curve is comprised of two components: the first reflecting the current passing through the ECF layer with little attenuation, ‘the shunting effect’; and the second reflecting the current passing through the homogenous brain tissue. However, when the ECF filled peri-electrode space is not present (dashed line) the curve through the tissue declines more rapidly over distance.

### 3.2 EBI with giant cell reactions

**Fig. 3:**
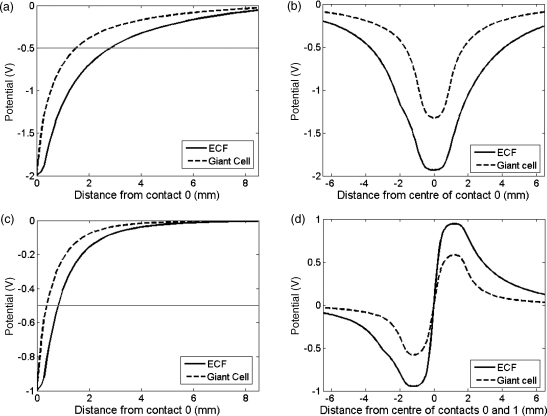
The figures show the difference between the cross-sections through the potential distributions created around the simulated electrode, when the electrode is surrounded either by giant cells or by ECF. Two stimulation setting are shown: monopolar −2 V stimulation via contact 0 (plots (a) and (b)) and bipolar ±1 V stimulation via contacts 0 and 1 (plots (c) and (d)), with a hypothetical activation threshold at −0.5 V (grey line). In (a) and (c), the fields are plotted as distance from contact surface radially outwards into the surrounding tissue, and in (b) and (d) distance is zeroed at the centre of the fields. In all cases, a larger magnitude of potential is consistently induced in the ECF case.

Giant cell formation alters the structure and the biophysical properties of the peri-electrode space, as the giant cell membrane has lower conductivity (0.125 S/m) than extracellular fluid. Fig. 3 shows cross-sections through the potential distribution of the model containing an ECF layer (acute), or a giant cell layer (chronic). Due to the shunting effect through the ECF-filled layer, and the shielding effect of the low-conductivity giant cells, the magnitude of the electric potential at a given distance is consistently less in the chronic case than in the acute case. This result is consistent for monopolar and bipolar stimulation settings, and for both directions measured. If we set a hypothetical activation threshold at −0.5 V, for example (dotted line in Fig. 3), there will be a difference in the effective range of activation between the ECF state and the giant cell state of ∼1.4 mm in distance during monopolar stimulation (Fig. 3a), and ∼0.4 mm in bipolar stimulation (Fig. 3c).

### 3.3 Modulation of brain pulsation on the EBI

As blood rhythmically perfuses into the brain, there is an increase in tissue conductance and an increase in the pressure on the electrode surface. During the acute stage, the thickness of the space around the implanted electrode may also be reduced. Over a heart beat cycle, the conductance of the surrounding brain tissue increases from 0.2000 S/m to a maximum of 0.2001 S/m. Meanwhile, the pressure on the electrode increases, and the thickness of the peri-electrode space decreases. Taken together, our simulations showed that the average induced potential in the surrounding tissue could increase by 0.34 ± 0.29% (mean ± standard deviation) during monopolar and by 0.41 ± 0.32% during bipolar stimulation. This suggested that physiological brain pulsation causes a measurable but small effect on the stimulation potential distribution in the surrounding tissue, which was increased during bipolar stimulation.

### 3.4 EBI with different electric fields in monopolar and bipolar settings

**Fig. 4:**
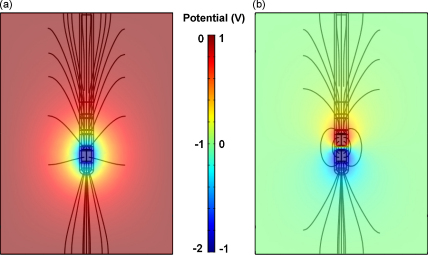
Electric field lines of (a) monopolar stimulation of −2 V via contact 0; (b) bipolar stimulation of ±1 V at contacts 0 and 1. Bipolar stimulation induces a near-field dipole around the two active contacts, whilst monopolar stimulation induces a far-field dipole between the single active contact and the IPG case.

The qualitative differences in the spatial distribution of the electric field were investigated further in three-dimensions and show that during monopolar stimulation, in which contact 0 is set at −2 V, the electric field spreads radially outwards from the active contact, which is consistent with the view that a ‘far-field dipole’ is created between the active contact and the case of the implantable pulse generator (IPG) (Fig. 4a).

For bipolar stimulation, both contacts 0 and 1 were activated at ±1 V, in order to maintain a comparable potential difference of 2 V. The field surrounding the electrode forms a typical ‘near-field dipole’ with the electric field centred around contacts 0 and 1 (Fig. 4b). These results indicated that bipolar stimulation has a smaller range of effect than monopolar stimulation. We quantified this difference by measuring the field at a distance of 2 mm from the electrode, in a perimeter around contact 0, and found that monopolar stimulation induced a field which was 50 ± 5% stronger than the bipolar field.

**Fig. 5:**
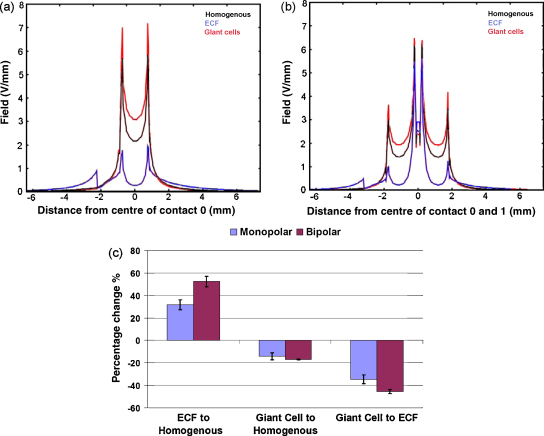
Comparison of electric field distribution measured axially at the interface between the peri-electrode space and the surrounding tissue during (a) monopolar and (b) bipolar stimulation. Three situations are shown: (i) with a layer of ECF in the peri-electrode space; (ii) with no peri-electrode space at all; (iii) when the space is filled by the growth of giant cells. The quantitative difference between the induced field strengths are shown in (c), for both monopolar and bipolar stimulation, in three cases: (i) homogenous tissue compared to including a ECF layer; (ii) homogenous tissue compared to including a layer of giant cells; (iii) comparing an ECF layer to a giant cell layer. These percentage changes show that the content effect of the peri-electrode space on the induced electric field is greater in bipolar stimulation than monopolar stimulation.

We quantified the percentage difference between cases for monopolar and bipolar settings. Fig. 5 shows measurements of the electric field distribution at the peri-electrode space and brain interface along the surface of the electrode, in three cases: (i) with no peri-electrode space, (ii) with ECF filled peri-electrode space and (iii) with giant cells. Our results showed that the inclusion of an ECF layer resulted in the field strength being underestimated by 37.2 ± 0.5% during monopolar stimulation and 58.2 ± 0.8% during bipolar stimulation (left-hand bar, Fig. 5c). Conversely, if giant cells were not modelled, the simulations overestimated the field strength by 14.1 ± 0.04% and 17.1 ± 0.04% (centre bar, Fig. 5c). Comparing acute to chronic stages, we found that inclusion of an ECF layer predicted a greater field strength than in the giant cell condition by 37.4 ± 0.3% and 47.7 ± 0.3% (*p* < 0.0001, *n* = 12) (right-hand bar, Fig. 5c). Our two-way ANOVA showed that these differences are consistently larger in bipolar setting than in monopolar setting (*p* < 0.0001, *n* = 12), and the changes in the field strength over conditions interacted with the electrode settings (*p* < 0.0001, *n* = 12).

## 4 Discussion

The present structural model of DBS was used to investigate an important issue of what effect the EBI has on the stimulation-induced potential distribution, focusing on the effect of the inclusion and modulation of the peri-electrode space. The injected current must pass through the peri-electrode space before reaching the surrounding tissue, however the effect of this interface was overlooked in several previous computational modelling studies [4–6,26]. We defined the biophysical properties of the model based on previous biological measurements, under three conditions *in vivo*: (i) acutely filled with extracellular fluid [24]; (ii) chronically filled by reactive giant cells [19]; (iii) dynamically modulated by brain pulsation [27]. Our results showed that in the acute stage following implantation, when the peri-electrode space is filled by extracellular fluid, this layer produces a ‘shunting effect’ as reflected by less attenuation of the electric field over distance compared with other conditions. In the chronic stage the properties of the peri-electrode space are altered by the growth of giant cells which we have shown produce a ‘shielding effect’ on the injected current passing through the EBI, i.e., the decline in potential intensity is more rapid than in the ECF case, and subsequently the volume of tissue activated will be significantly reduced. This confirms the clinical observation that the therapeutic effects of DBS may gradually become less effective over time. One may argue that the overall stable clinical benefit of DBS for years after implantation suggests that the significance of giant cell formation is limited. However, we would like to point out that the impact of the giant cell growth on the current distribution may be profound in the short period of weeks to months following implantation, as there is often a need to gradually increase the intensity of the stimulation. In this case, although the therapeutic effect is maintained, the energy consumption is increased so that the life expectancy of the stimulator battery is reduced and battery replacement becomes more frequent. This change cannot be associated with the progression of the disease, but as our study suggests may be due to factors such as giant cell growth at the interface. This can be validated by accurate impedance measurements of the EBI during the transition from the acute to the chronic stages post-implantation.

In comparison with giant cell growth over a period of weeks, the physiological modulation of the EBI due to brain pulsation occurs over a much smaller timescale of seconds. Our results showed that the brain pulsation had a measurable but small effect on the potential distribution in the surrounding tissue. To fully evaluate the significance of this small scale modulation under physiological conditions, the following factors need to be taken into consideration: (1) The small modulation values over a cardiac cycle were likely to be underestimated as only the resistive, not the time-dependent reactive, component of the EBI was simulated due to the static nature of the current structural model; (2) though the impact on the large amplitude stimulation current may be small, this may have more profound effects on the accurate amplitude measurement of local field potentials of a few μV crossing the EBI in the opposite direction [27]; (3) the electrical charges generated by the metal electrode and the ECF may be further polarised by the injected electrical current during deep brain stimulation in the acute stage. A recent study [22] showed that in acute subthalamic stimulation for Parkinson's disease, the magnitude of the low-frequency electrode potential gradually declined over a period of minutes after the stimulation ceased, which correlated with the gradual reappearance of symptoms.

### 4.1 Computational considerations

As with any computational modelling study, the assumptions made and the scope of the work must be considered carefully. For example the current model investigated the potential distribution induced by DBS via the solution of Poisson's equation over a three-dimensional structure. However, a clear shortcoming of this model is that there is no temporal aspect to the solution. Previous work has utilised a Fourier-FEM method for introducing time dependence into such models [5], and has shown that consideration of time-dependence and the shape of the stimulus waveform allows a more accurate estimation of the volume of tissue activated by DBS (by 20%). Other studies have considered the effect of the stimulus waveform, as well as the tissue properties on the electrode impedance which is measured intra-operatively [4]. In the current work, we used a quasi-static approximation to look at the field shape created, and the percentage difference between the potential distributions created in different stimulation paradigms. Therefore, as we did not explicitly comment on the absolute range of the field, the use of quasi-static approximation is unlikely to have affected our conclusions. Future work should extend the current approach in order to account for the time dependence of the stimulation current, as well as the electrode impedance changes by coupling a time-dependent FEM model to a complete electrode model of the instrumentation.

Another way to extend the mathematical approach used in our current study would be to utilise a combined boundary element method (BEM) and FEM technique to deal with the thin shells. For example, recent work of a shell model of the whole head highlighted the limitations of a FEM approach for solving models which contain multiple thin layers [16]. In the previous case the author focused on the scalp, skull, CSF, etc., whereas in the present case the thin layer of peri-electrode space surrounding the electrode may be a source of error for the results. However, such errors are not likely to influence our conclusions, as we have looked at the relative differences between different conditions.

An important issue regarding the approach taken in this study is why a FEM solution was simulated rather than finding an analytical solution of Poisson's equation? By definition, the finite element method is an approximation of the precise solution to a partial differential equation over a specified region. Poisson's equation can be solved analytically, and therefore precisely for a shelled cylinder. Therefore, it would be possible to approximate the structure of the EBI defined here by a shelled cylinder, and solve precisely for this structure. However, making such an approximation would still result in an error in the calculated potential distribution induced by DBS. Once more, as we are concerned with the relative differences between stimulation paradigms, the errors introduced by FEM become less significant.

### 4.2 Interaction between EBI and electrode configuration

The multi-contact electrodes used in neurostimulation can be configured to match the contact and target positions without the need to surgically change electrode location. We investigated the effect of the content of the peri-electrode space on such contact configurations and their electric potential distributions using our EBI model. We demonstrated that monopolar stimulation produced a long-field dipole with a more far-reaching and quantifiably stronger potential distribution, compared to the small-field dipole induced by the bipolar stimulation (Fig. 4). Recent studies support this result [9] by showing that bipolar stimulation does not cause electrical artefacts during routine electrodiagnostic procedures whereas monopolar stimulation does; and calculations of the distance of maximum activation produced by monopolar and bipolar stimulation settings with equal intensity, found the difference to be up to 3.9 mm. Therefore, when considering the size and shape of the target nucleus, in addition to adjusting the current intensity to control the volume of brain tissue being activated, the selection of monopolar or bipolar settings may also be helpful in optimising the therapeutic stimulation and reducing unwanted side effects. It is important to note however, that the IPG sets the voltage with a reference at either a nearby contact (bipolar), or the IPG case (monopolar). Therefore, in addition to the differential potential, the absolute voltage of each activated contact needs to be taken into consideration. These results may help to explain the clinical observation that, to maximally stimulate the globus pallidus interna, bipolar stimulation via contacts 0 and 3, or monopolar stimulation via contact 0 are optimal; whereas monopolar stimulation via contact 3 could also minimise tremor (though not as well), but caused more abnormal involuntary movements [20]. Another recent study on the direct effect of subthalamic nucleus stimulation on levodopa-induced peak-dose dyskinesia in patients with Parkinson's disease [14], suggests that bipolar STN-DBS with contacts placed above the STN used as an anode appears to represent a useful option for simultaneously controlling both the cardinal symptoms of PD and dyskinesia.

In the present study, we revealed the effects of including an ECF filled peri-electrode space, and of modelling the growth of giant cells on the two stimulation paradigms. In both monopolar and bipolar settings neglecting the ECF filled peri-electrode space leads to underestimating the field strength induced, whereas ignoring the growth of giant cells results in an overestimation. We found that the effect of ECF presence and giant cell growth is more pronounced in bipolar than in monopolar settings. This is intuitive based on the knowledge that the electric field created in bipolar stimulation is a small field dipole, and therefore changes at the peri-electrode space are able to distort the field. Conversely, monopolar stimulation creates a large distance dipole, and therefore local changes at the peri-electrode space have less impact on the field shape and strength. These results indicate that the inclusion of the peri-electrode space is essential to accurately predicting the electric field shape and strength in simulations of the implanted DBS electrode.

### 4.3 Implications and conclusions

This study is largely based on the currently used implantable pulse generator, which is a voltage-controlled device, and the electrostatic model presented here simulates such voltage-controlled stimulation. With such a device the size of brain volume being activated is highly dependent on the tissue conductivity, and as the conductivity decreases during the transition from the acute to the chronic stage, the amount of current spread into brain tissue also decreases. This electrostatic structural model may serve as a base for future study of current-controlled pulse generators, as changes in the tissue conductivity are automatically compensated by the stimulator, therefore maintaining constant activation despite the changes at the EBI.

The structural model presented here replicates experimental evidence showing that the shape and extent of the electric field created by DBS is modulated by physiological and pathological factors affecting the EBI, which in turn influences the configuration of the multi-contact electrode. In particular, we have shown that the field induced by the same intensity of stimulation during acute stages is stronger than that induced in the chronic stage. This is due to both the shunting effect through the high-conductivity ECF layer, and the shielding effect of the low-conductivity giant cells. This has implications for prediction of current settings made based on such models at these different stages post-implantation. Furthermore, these differences are more significant for the near-field dipoles induced by bipolar stimulation than the far-field dipoles of monopolar stimulation. Therefore, the computational approach and the results of this study should aid to achieve a better understanding of the spread of current induced by DBS. Further modelling work aims to make predictions for stimulation paradigms by taking the clinical situation of individual patients into consideration.

## Conflict of interest

The authors have no reported conflict of interest.
